# Development and Effectiveness of a Patient Safety Education Program for Inpatients

**DOI:** 10.3390/ijerph18063262

**Published:** 2021-03-22

**Authors:** Sun Hwa Shin, Mi Jung Kim, Ho Jin Moon, Eun Hye Lee

**Affiliations:** 1 College of Nursing, Sahmyook University, Seoul 01795, Korea; shinsh@syu.ac.kr; 2 Sahmyook Medical Center, Seoul 02500, Korea; sabin22@hanmail.net (M.J.K.); lura74@hanmail.net (H.J.M.)

**Keywords:** patient safety, quality improvement, education, inpatient

## Abstract

Background: Patient safety is considered an important issue in the field of healthcare, and most advanced countries. Purpose: This study was designed to evaluate a patient safety education program among hospitalized patients. Of the 69 participants, 33 completed the patient safety education program while the 36 remaining participants were given educational booklets. The program was used to measure knowledge about patient safety, patient safety perception, and willingness to participate in patient safety. Methods: Patient safety education was developed by the analysis–design–development–implementation–evaluation model considering expert advice, patient needs, and an extensive literature review. Data were collected from 20 July to 13 November 2020. Data were analyzed using SPSS statistical program. The effectiveness of the experimental and control groups before and after education was analyzed using paired *t*-tests, and the difference in the amount of increase in the measured variables for each group was analyzed using independent *t*-tests. Results: The experimental group had significantly higher patient safety scores (*t* = 2.52, *p* = 0.014) and patient safety perception (*t* = 2.09, *p* = 0.040) than those of the control group. However, there was no significant difference between the two groups regarding the willingness to participate in patient safety. Conclusion: The patient safety education program developed using mobile tablet PCs could be an effective tool to enhance patient involvement in preventing events that may threaten the safety of patients. Further studies are recommended to develop a variety of educational interventions to increase patient safety knowledge and perceptions of patients and caregivers.

## 1. Introduction

The importance of “patient safety” has been emphasized ever since the Institute of Medicine (IOM) reported that inpatients are more likely to die from adverse events than by traffic accidents [1,2,3]. Currently, patient safety is considered an important issue in the field of healthcare, and most advanced countries have not only introduced patient safety reporting systems to prevent the recurrence of adverse events but also prepared related laws to establish a patient safety management system [4,5]. Even in Korea, multidimensional efforts have been made based on the perspective of medical and healthcare providers in order to prevent medical malpractices, medical errors, and adverse events by fostering patient safety culture, implementing a healthcare accreditation system, and introducing patient safety education and prevention activities [6].

Patient safety refers to a state in which a patient’s own safety is not threatened [7], suggesting that the patient’s active participation in patient safety activities is the best way to reduce medical accidents [8,9]. In order to prevent errors that may occur while treating patients, “patient-centered medical care” can be realized by strengthening obligations of the healthcare providers and legal regulations for patient safety and establishing a patient safety culture [10,11]. While the patient safety activities centered on healthcare providers have been the main focus of research, recent emphasis has been on the participation of patients and caregivers, who are the healthcare consumers [8,12], in patient safety activities.

The subjects of most previous studies on patient safety educational programs were medical students [13,14,15], nursing students [16,17], and healthcare providers including doctors and nurses [18,19,20]. In particular, research was conducted on a group of nurses who were acutely aware of various issues related to patient safety in the medical environment. In some studies, patient safety education was delivered using data from the “Participate Inform Notice Know (PINK)” video for inpatients [21,22], wherein education on patient safety was imparted using audiovisual educational materials [23], smartphone videos [24], and a smartphone application [25] for surgery patients. However, rather than dealing with the overall contents of patient safety, the training focused on precautions, fall prevention, and postoperative self-management in accordance with the characteristics of the patient’s disease [24,25,26,27]. In addition, the U.S. holds a campaign to allow patients to participate in patient safety through “speak-up” activities centered on medical institutions and actively report accidents to prevent patient safety accidents [28]. However, in Korea, there are no national campaigns as well as studies that have verified the effectiveness of patient safety education conducted on patients and caregivers. Thus, it is important for patients to have an active willingness to participate, in order to secure their own safety; this is the direction in which patient safety education for patients should proceed [29]. Recently, patient safety education, which was limited to patient safety officers and healthcare providers in hospitals, has been expanded to target patients and caregivers; however, only a few studies have determined the effects of patient safety education on patients or caregivers [30].

In prior studies, patient safety education improved patient safety knowledge [13,25,29] and patient safety perception of subjects [29] and increased communication skills [19]. Inpatients who watched PINK videos increased their comfort in participating in patient safety activities [21], and changed their attitudes and capabilities in patient safety. As such, patient safety education will increase the willingness to participate by inducing improvements in knowledge and awareness and changes in attitudes [13,14,17,31] The willingness to participate in patient safety refers to the practice of direct health behavior, so it needs to be considered as an important variable to examine the effectiveness of patient safety education.

Currently, education on fall and infection prevention is promoted mainly through printed materials for patients and caregivers in domestic hospitals. From the perspective of long-term memory according to the educational method, the approach that utilizes video with audiovisual elements is said to be more effective in terms of learning [32]. Likewise, it is necessary to visualize and present information on patient safety in various ways through mobile images in order to increase its educational effect. It is also important to study the educational medium and utilization method with enhanced accessibility to effectively impart patient safety education to the general public. At the time when non-face-to-face education is being activated due to the COVID-19 situation, developing a mobile-based patient safety education program will increase the use of education not only for hospital inpatients but also for targets in the community. This study developed a patient safety education program for inpatients admitted to general hospitals and implemented it by utilizing a tablet PC to examine its effectiveness.

The research questions derived from this study are as follows: 1. Does patient safety education increase patient safety knowledge of inpatients? 2. Does patient safety education increase patient safety awareness of inpatients? 3. Does patient safety education increase the willingness of inpatients to participate in patient safety?

## 2. Methods

**Figure 1:**
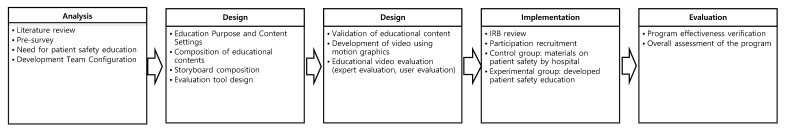
Analysis–design–development–implementation–evaluation (ADDIE) application for program development in this study.

In this study, an instructional systems design was employed based on the analysis–design–development–implementation–evaluation (ADDIE) model [33] to develop a patient safety education program (Figure 1). According to the procedure of the ADDIE teaching–learning model, the literature on patient safety education related to learning was reviewed and the needs of the subjects were researched and analyzed. After the educational contents were designed, based on the analysis outcomes, and the teaching strategy was established, audiovisual learning materials using motion graphics were developed. After implementing the education program developed for inpatients, its effects on the knowledge, awareness, and willingness to participate in patient safety were measured.

## 3. Development of the Patient Safety Education Program

### 3.1. Step 1: Literature Review and Pre-Survey

The literature review on patient safety revealed that patient safety education was provided mainly to healthcare providers, implying that there were few studies that developed a patient safety education program targeting general patients; the effects of such education programs were examined [6,8,14]. In addition, because specific information for patients or caregivers cannot be provided in an integrated manner since patient safety education is imparted based on the circumstances of each hospital, the need to provide such information has been highlighted [30]. Based on the analysis of the literature review, the need for patient safety education was investigated for the general public [34]. A panel survey was conducted by EMBRAIN (Korean survey company) from 13 to 27 November 2019, among 1187 respondents who visited the hospital within one year from the time of the survey.

There were 591 male participants (49.8%) and 596 female participants (50.2%), with an average age of 44.3 years (±13.1). The survey revealed that 1138 people (95.9%) answered that patient safety education was necessary. With regard to the patient safety education requirements, 1172 respondents (98.7%) wanted content on “coping method in case of patient accidents”; 1167 (98.3%) wanted content on “types of patient accidents in hospitals”; 1157 (97.5%) wanted content on “medical information communication and decision-making method”; 1145 (96.5%) wanted content on “types of patient safety activities conducted in hospitals”; 1136 (95.7%) wanted content on “patient safety laws”; 1133 (95.5%) wanted content on “participation in patient safety activities”; and 1054 (88.8%) wanted content on “healthcare accreditation system.” These results revealed that most participants acknowledged that patient safety education was essential [34].

### 3.2. Step 2: Development of Patient Safety Education Program for Inpatients

#### 3.2.1. Composition of Educational Contents

For content development of the patient safety education program, a list of contents was created based on the findings of the literature review and pre-examination. Content validity was assessed by field professionals including five current nurses, four head nurses, three nurses dedicated to quality management, and two professors of nursing. The average age of these professionals was 42.4 (±7.07) years; six were university graduates with a bachelor’s degree, while eight had a master’s degree. Regarding their clinical experience, one person had 3–5 years of work experience, three people had 6–9 years of work experience, and ten people had 10 or more years of work experience. All had prior experience in providing patient safety education for inpatients. Their educational methods varied with 11 providing printed materials, one making use of videos, and six imparting one-on-one education. Thus, imparting education on patient safety was mostly conducted through printed materials. The validity of the educational contents and organization was assessed using a 4-point scale, ranging from “very appropriate” (4 points) to “very inappropriate” (1 point). Appropriateness was assessed by screening contents with a value of 0.80 in the item-content validity index (I-CVI). Regarding the contents of patient safety education, because the Five Rights of Medication Administration are applicable only to healthcare providers, it is inappropriate for the general public. The inspection of medical equipment and instruments was also inappropriate because it would be difficult for inpatients to check these in reality. Eventually, the educational contents were revised and supplemented by collecting additional opinions from professionals. The final educational contents selected for patient safety consisted mainly of four items: understanding of patient safety; introduction of patient safety activities; patient safety participation method (accurate communication, activities to prevent falls, activities to prevent infection, activities to prevent bedsores); and how to deal with a patient’s accidents.

#### 3.2.2. Development of Video Using Motion Graphics

Educational media on patient safety was produced in a video using motion graphics. After developing a scenario focusing on the contents of patient safety education, the scenario was revised under the supervision of three professionals (two nurses dedicated to quality management and one professor of nursing). The texts and content composition were revised by a field professional with experience in video production. The animation was produced by a motion graphic production company, and an announcer was recruited to record the voice. The video was produced using storyboard composition for each topic. Two types of videos comprised the patient safety education program, as shown in Table 1. The running time of Video 1 was 7 min and 38 s; it comprised contents related to understanding of patient safety, patient safety activities, and participation in the patient safety program. The running time of Video 2 was 7 min and 6 s; it was about reviewing the activities to prevent falls, infection, and bedsores and how to participate in the patient safety program.

### 3.3. Step 3: Evaluation of the Video Education Program

#### 3.3.1. Professional Evaluation

The completed educational video was evaluated by 11 professionals (five nurses dedicated to quality management, three nurse managers, and three head nurses). The average age of the professionals was 48.4 (±7.59) years; eight individuals had a master’s degree and three had doctorates. Two had less than 10 years of clinical experience while nine people had over 10 years of work experience. The professional evaluation of the videos was scored on a 4-point scale ranging from “strongly disagree” (1 point) to “strongly agree” (4 points) in terms of accuracy (four items), benefit (six items), and comprehensibility (three items). Accuracy was 3.54 to 3.82 points on average, which implied that the videos accurately delivered information on patient safety and did not contain medically erroneous information. The average score for benefit was 3.63 to 3.82 points, which implied that the videos contained useful information on patient safety for the general public without exaggerated contents and did not contain triggers of harmful behaviors to individuals. The average score on comprehensibility was 3.27 to 3.73 points, with the professionals evaluating the contents to be adequate for the general public, including the elderly, to easily understand the use of the official medical terminology. The educational contents of the videos were evaluated to be interestingly organized, but some pointed out the necessity for improvement as the scenes shifted rapidly, the voice actors spoke quickly, and the running time was long due to inclusion of many contents. Accordingly, the nurse character created to impart patient safety education was modified to reflect the professionals’ opinions, and the clothes of the patients and hospital staff were revised so they could be easily distinguished. Additionally, the texts, conversation, and narration speed of the video were supplemented.

#### 3.3.2. User Evaluation

The comprehensibility of the modified videos (three items) was evaluated one by one by four individuals from each age group including teenagers as well as those in their 40 s, 50 s, and 60 s. The average score of the comprehensibility was 3.09 to 3.46 points, which implied that the videos were easy to understand and provided useful information.

## 4. Effectiveness of the Patient Safety Education Program

### 4.1. Study Design

This study is similar to an experimental study that aims to verify the effect of a patient safety education program using a tablet PC on patient safety knowledge, patient safety awareness, and patient safety participation intention for hospitalized patients in general wards using nonequivalent control group pre-posttest non-synchronized design (Table 2).

### 4.2. Participants

The subjects of the study were adults over 20 years of age who were admitted to the general ward at S Hospital in Seoul. These patients were admitted to the three wards included in the study (two internal medicine wards and one surgical ward). The selection criteria for the subjects were: patients over 20 years old; patients admitted in a general ward; patients who were scheduled to be hospitalized for more than one week; and those who could understand and respond to the questionnaire without cognitive dysfunction. Meanwhile, persons under the age of 20, patients admitted to the intensive care unit, those who were scheduled for short-term hospitalization for special treatment purposes (within three days), and those who could not understand the questionnaire due to cognitive dysfunction were excluded from the study.

**Figure 2:**
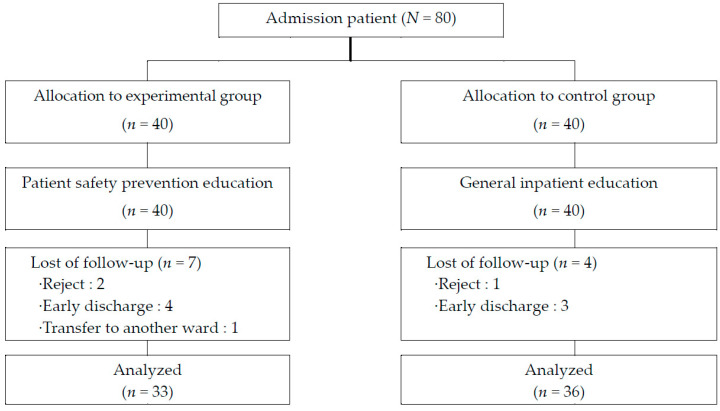
Flow diagram of study participants.

The number of subjects in this study was calculated using G-power 3.1.9.2 (Heinrich-Heine-Universität, Düsseldorf, Germany). According to the calculation results obtained with values of 0.50 for the effect size, 0.05 for the significance level, and 0.80 for the power based on the paired t-test, the number of the subjects was calculated as 34 individuals for each group. In consideration of the drop-out rate, a total of 80 individuals were selected (40 per group), and the subjects were recruited during the study period. The number of samples was calculated through G-power verification during the research plan, and IRB approval was obtained from the relevant medical institution for the selection of research participants. The wards for which the study was conducted were selected as internal medicine and surgical wards with many inpatients for more than one week. The purpose of the study was explained to the subjects who expressed their intention to participate in the study during the hospitalization period through the recruitment announcement for research participation, and the research assistant received the consent form and screened whether it was suitable for the study. The main reasons for dropping out of the subject were early discharge, transferring other wards or hospitals. Researchers and research assistants conducted research on ethical principles, and three to four nurses participated in the study after receiving training on research purposes and patient safety education in advance in the ward. After conducting a preliminary survey on the day of hospitalization, we trained the use of the tablet PC for patient safety education, and visited the target person to repeatedly watch the training video on the 3rd and 5th day of hospitalization. After conducting a follow-up survey on the 7th day of hospitalization, we provided a reimbursement. Finally, patients who were hospitalized from 20 July to 11 September 2020 were assigned to the control group while those admitted from 20 September to 13 November 2020 were assigned to the experimental group. In the course of the study, one individual who refused to answer the questionnaire and three who were discharged early were dropped from the 40 subjects in the control group. Likewise, two who refused to answer the questionnaire, four who were discharged early, and one who was transferred to another ward were dropped from the experimental group. Therefore, the data from a total of 33 and 36 individuals in the experimental and control groups, respectively, were used for the final analysis (Figure 2).

### 4.3. Measurements

#### 4.3.1. Patient Safety Knowledge

The questionnaire was developed based on the cognitive knowledge items on patient safety presented in the studies of Lee [35] and An et al. [29], validated with content validity verification for measuring patient safety knowledge. The patient safety knowledge instrument consists of 31 questions, including the definition of patient safety accidents (3 items), report in case of patient safety incidents (3 items), role of patient (3 items), fall prevention (3 items), what patients need to know when taking drugs and getting injections (6 items), what the patient needs to know during the procedure (3 items), patient identification procedure (5 items), and hand hygiene (5 items). Correct answers to each question are given 1 point, while wrong answers are scored 0; the total possible score is 31 points. Higher scores indicate higher patient safety knowledge. The reliability of the tool in this study was 0.73 in Kuder–Richardson Formula 20 (KR-20).

#### 4.3.2. Patient Safety Perception

Patient safety perception was measured using a patient safety perception instrument developed by Kim, Lee, and Shin [36] for inpatients in Korea, based on the Patient Measure of Safety (PMOS) by Giles, Lawton, Din, and McEachan [37] for inpatients. The measuring instrument was used after obtaining the permission of the original author. The patient safety perception tool developed preliminary questions after verification of the content validity by experts at the time of development, and through the survey, it verified the construction feasibility, the referenced feasibility, and the reliability. It consists of 24 questions and is composed of the subfactors of safety assurance activities (10 items), safety practice (10 items), and trust in the medical system (4 items). Each item is rated on a 5-point Likert scale, ranging from “very much” (5 points) to “not at all” (1 point). Higher total scores reflect greater perception of patient safety. In the study by Kim et al. [36], the reliability of the tool at the time of development, as measured by Cronbach’s α was 0.93; in this study, it was 0.98.

#### 4.3.3. Patient Participation Willingness

Patient participation willingness was measured using the measurement tool developed by Lee [38]. The measuring instrument was used after obtaining the permission of the original author. The willingness to participate in patient safety was verified by experts for content validity for the developed items. The willingness to participate in patient safety consists of 18 questions and 5 subfactors (decision making, information provision, questioning, confirmation, and reporting). Each item is rated on a 4 point Likert scale, ranging from “very much” (4 points) to “not at all” (1 point). Higher total scores reflect greater willingness to participate in patient safety. In Lee’s [38] study, the reliability of the tool, as measured by Cronbach’s α was 0.88; in this study, it was 0.97.

### 4.4. Data Collection and Procedure

To prevent the spread of treatment effects between experimental and control groups, the study applied different data collection periods. One week after completing the control data collection, the experimental group’s data collection proceeded. Data of the control group was collected from 20 July to 11 September 2020, and the data of the experimental group was collected from 20 September to 13 November 2020. In the process of data collection, 11 nurses working in the three wards and a nurse working in the Quality Improvement Department participated in the study as research assistants. They were given prior information about the purpose, procedures, and methods of data collection of this study. A notice for study participation was posted in the wards and, after confirming the intention of the inpatients about study participation during the research period, a description of the study participants was presented. The research proceeded after the subjects read the description and signed the consent for study participation.

A pre-survey was conducted on the first day of the patients’ admission for both the experimental and control groups. The general characteristics, patient safety knowledge, patient safety awareness, and intention regarding patient safety participation were assessed. After conducting the pre-survey, the nurse in charge provided the control group with not only hospitalization instructions produced by the hospital but also education materials on patient safety. Meanwhile for the experimental group, the research assistants promoted the developed patient safety education three times. After conducting the pre-survey on the first day of the patients’ admission, the first educational session on patient safety was conducted utilizing a tablet PC. The second video on patient safety was shown on the third day of hospitalization. On the fifth day of hospitalization, the subjects were asked to recall the content from the videos shown during the first and second educational sessions. For the post-survey, the research assistants conducted a survey on the same variables as in the pre-survey. The post-survey of the control group was carried out five to seven days after the admission while that of the experimental group was conducted on the seventh day of hospitalization (Table 2).

### 4.5. Data Analysis

The collected data related to the effect of patient safety education were analyzed using the SPSS/WIN 25.0 statistical program. The general characteristics of the subjects were analyzed by frequency, percentage, mean, and standard deviation, and the prior-homogeneity verification of the experimental and control groups was analyzed by the X^2^ test and the independent *t*-test. The effectiveness of the experimental and control groups before and after education was analyzed using paired *t*-tests, and the difference in the amount of increase in the measured variables for each group was analyzed using independent *t*-tests. All statistical significance levels were set to 0.05.

### 4.6. Ethical Considerations

All subjects gave their informed consent for inclusion before they participated in the study. The study was conducted in accordance with the Declaration of Helsinki. Prior to the data collection, research approval was obtained from the Institutional Review Board (IRB No: 116286-202005-HR-01) of S Hospital in June 2020. The purpose and methodology of the study were explained to the subjects, including matters related to confidentiality of their information, description of voluntary participation, and their right to stop participating at any time. They were also assured that they would incur no disadvantage by participating in the study. The collected survey data was anonymized, then coded and analyzed; after saving the data, the questionnaires were stored and locked in a safe place. The subjects who participated in the study were each provided with a gift certificate after completing the pre- and post-survey. After completing the post-survey, the patient safety education that was offered to the experimental group was imparted to the control group as well in consideration of ethical aspects.

## 5. Results

### 5.1. Verification of the Sameness of the Subjects

The general characteristics of the subjects are represented in Table 3. Of the 69 total subjects, 28 (42.0%) were in their 60s or older and 39 (56.5%) were male. Regarding academic background 26 (37.7%) were high school graduates and 24 (34.8%) were university graduates. As for the economic level, the middle-class accounted for the most at 40 (58.0%). Among the hospitalized patients, 46 (66.7%) had no religion. There was a large distribution of 47 (68.1%) who had jobs. With regard to their health, 43 (62.3%) had no underlying disease, and 37 (53.6%) had an average frequency of less than one visit per year. A large proportion of participants—45 (65.2%)—underwent surgery, more than those who did not. As for the participant’s medical department, 41 (59.4%) internal medicine as many. There were 55 patients (79.7%) who had experience in patient safety education; 35 patients (43.8%) with the type of prior patient safety education and 27 patients (33.8%) received handouts of medical information.

The pre-identity verification of the experimental (*n* = 33) and control (*n* = 36) groups revealed no significant difference between the two groups (*p* > 0.05), and the homogeneity was secured. The duration of stay in hospitalization of the experimental group was at least 7 days, maximum of 39 days, and average 10.85 (±8.20) days, and the duration of stay in hospitalization of the control group was at least 5 days, maximum of 41 days, and average 8.92 (±4.29) days. In addition, the average score of the two groups for the main measurement variables were compared: patient safety knowledge (*t* = −1.68, *p* = 0.099), patient safety perception (*t* = −0.99, *p* = 0.327), and willingness to participate in patient safety (*t* = 0.15, *p* = 0.882); because there was no significant difference in willingness, they were homogeneous (Table 3).

### 5.2. Validating the Effectiveness of the Patient Safety Education Program

In order to analyze the effect of the patient safety education program for inpatients, the results of analyzing the difference in the amount of increase in the measured variables by the two groups are represented in Table 4.

After application of the patient safety education program, the patient safety knowledge score of the experimental group (2.36 ± 3.44) was higher than that of the control group (0.56 ± 2.48), and the difference in the amount of increase between the two groups was significant (*t* = 2.52, *p* = 0.014). In patient safety knowledge, the experimental group significantly increased the post-score compared to the pre-score (*t* = 3.94 and *p* < 0.001), the control group showed no significant increase (*t* = 1.35, *p* = 0.187).

After application of the patient safety education program, the patient safety perception score (11.79 ± 17.95) of the experimental group was higher than that of the control group (3.17 ± 16.27), and the difference in the amount of increase between the two groups was significant (*t* = 2.09, *p* = 0.040). In patient safety perception, the experimental group significantly increased the post-score compared to the pre-score (*t* = 3.33, *p* = 0.002), the control group showed no significant increase (*t* = 1.17, *p* = 0.251).

After application of the patient safety education program, the willingness to participate in patient safety score (4.64 ± 7.99) of the experimental group was higher than that of the control group (3.28 ± 7.83), but the difference in the amount of increase between the two groups was not significant (*t* = 0.71, *p* = 0.478). Subject’s willingness to participate to patient significantly increased post-scores compared to pre-scores in the experimental group (*t* = 3.78, *p* = 0.001), the control group also saw a significant increase in post-score (*t* = 2.51, *p* = 0.017).

## 6. Discussion

The experimental group that participated in the patient safety education program developed in this study showed significantly higher scores on knowledge and awareness of patient safety than did the control group. However, it is necessary for patient safety education programs for inpatients be conducted repeatedly and for the motivation of the inpatients to participate in patient safety activities to be improved.

In this study, an educational video was produced using motion graphics, and patient safety education was imparted using a tablet PC. In previous studies, education on patient safety and fall prevention was promoted by utilizing videos and instructor-led education [22,29], mobile devices such as smartphones or tablet PCs [24,25], and simulation with cramming education on theories [39]. The patient safety education programs in the previous studies were mainly applied once for inpatients [22,24] and the educational contents were limited to fall prevention [40] or operation-related adverse events [25]. The patient safety program developed in this study included multidimensional patient safety activities on patient identification as well as prevention of infections, bedsores, and fires. This study differed from previous studies in that the contents were organized by focusing on the patients’ participation method. In addition, the study attempted to supplement the limitations of conventional education, imparted orally and through printed materials, by making the patients repeatedly watch the videos while in bed, and not by offering a one-time education session. Nevertheless, there was feedback that the running time of the videos was too long, which made it hard for the patients to concentrate on the contents. The total length of the video was about 7 min, as it included detailed information on patient safety. From the perspective of the information provider, the video material was sufficient for patient to easily utilize information; however, it may not be effective if the contents and length of the video made it difficult for the patients and caregivers to fully concentrate [41,42]. Therefore, it will be necessary to address such concerns in the future.

When it comes to applying the education intervention to preventing falls and bedsores among the patients, it is necessary to consider the time of the education program, severity of illness of the inpatient, presence of pain, and surgery status. In the two internal medical wards that were included in the study, many patients were hospitalized due to digestive, respiratory, and endocrine problems, while most patients in the surgical wards were orthopedic patients who were expecting to undergo surgery. The subjects admitted to the internal medicine ward tended to be unable to focus on the patient safety education due to acute pain and being subjected to various examinations conducted on the day of admission. Some patients admitted to the surgical ward were not able to concentrate on the patient safety education program on the day of admission because they were preparing to undergo surgery and on the third day due to postoperative pain. In a previous study, it was reported that inpatients received too much patient safety education in the hospital and because they were not interested, they did not go through the educational materials despite the hospital having invested in them at a high cost [41]. In addition, the inpatients tended to focus only on the patient education that was essential to the purpose of their admission to the hospital [42,43]. Patient safety activities are aimed at preventing all kinds of risks, errors, harms, and mistakes that may affect the patients [44], and the subject of these activities should be the patients. However, the actual inpatients were found to concentrate only on their own disease and not on patient safety. In addition, falls or bedsores are important issues for the elderly patients or those who have been bedridden for a long time due to limited body movement. Therefore, young patients or patients with no restrictions on body movement showed no interest in fall or bedsore prevention. Ever since the healthcare accreditation system was introduced in Korea, hospitals have been conducting various patient safety events. According to the results of related studies, patient safety education for patients and their caregivers had a short intervention effect, while education through new educational media and methods only showed a temporary effect [41]. It has been emphasized that active participation of patients and caregivers in patient safety activities is the best way to reduce medical accidents [8,45]. However, education and promotion of patient safety for general patients and caregivers are insufficient compared to the concern for patient safety in hospitals [29]. Since the nationally standardized education materials on patient safety are not currently available, there may be differences in the patient safety education programs imparted by each medical institution [41]. Moreover, it is necessary to standardize concise educational materials that can be easily understood by patients and caregivers, as the education provided by nurses in charge for patient safety are not persistent when patients are being frequently admitted and discharged [41]. In addition, it is important to develop a customized education program to enable the patients to concentrate by differentiating the contents and timing of the delivery of patient safety information according to the characteristics and severity of the patient’s disease.

The experimental group that participated in the patient safety education program improved their knowledge on patient safety. This finding was consistent with the results of previous studies that showed improved knowledge on patient safety after providing education to inpatients [25,29,39,46]. However, it is difficult to directly compare the results due to the absence of a standardized instrument that measures knowledge on patient safety for inpatients. The results of this study were also consistent with the results of previous studies that showed improved patient safety knowledge and educational satisfaction after repeated education sessions [24]. Providing an effective patient safety education program implied increased capability among the patients to avoid various accidents that may occur to them [41]. In order to increase the patients’ capability, it is basically necessary to provide correct knowledge on patient safety. Recently, various studies on patient education have been actively conducted to assess educational methods based on information and communication technologies such as virtual/augmented reality and functional games. In consideration of such a methodological approach, it is necessary to conduct research that can improve the patients’ capability by applying a variety of educational media to effectively increase patient safety knowledge among non-medical personnel.

The experimental group that participated in the patient safety education program improved their perception on patient safety after their participation. This finding was supported by the results of previous studies that showed improved awareness on patient safety among inpatients after imparting education by utilizing video and printed materials [29] and simulating situations [39]. With regard to patient safety education promoted for inpatients as well as the general public in the community, the overall awareness on patient safety improved [47]. Additionally, reinforcement of patient safety education by medical personnel will be more helpful in improving the perception of patient safety among inpatients [40,48]. Therefore, it is necessary to expand the perception on patient safety among the patients to ensure their own safety, actively examine patient safety activities, and communicate well with healthcare providers. Moreover, perception on patient safety will be enhanced by promoting it in the community, not just limiting it to hospitals, and by establishing an education system.

After the education program, the willingness to participate in patient safety activities increased in both the experimental group that participated and the control group that did not participate. Previous studies reported that a sense of comfort was improved among inpatients who watched the ‘“PINK” patient safety video [22]. Additionally, in a previous study, the awareness of participating in a patient safety program was enhanced after a two-hour lecture-style instruction on patient safety was imparted to 25 people [47]. These results reflect the results of this study. The PINK patient safety video was effective in encouraging patients to participate in patient safety activities such as checking the hand hygiene status of doctors or nurses, knowing their medications and allergies, and reporting errors [22]. In a previous study conducted in Korea, the inpatients’ willingness to participate in the patient safety activities such as checking the hand hygiene status of the healthcare providers and accuracy of the medicines provided by the healthcare providers were shown to be low [8,49] In situations with limited proactive communication between the healthcare providers and patients, there are limitations in the patients’ ability to assess the capability of the healthcare providers regarding their patient safety activities. According to the systematic literature review, the factors affecting the patients’ willingness to participate in patient safety were classified into disease, patients’ cognitive characteristics, relationship between patients and healthcare providers, and the hospital system [50]. Therefore, it is necessary to change the sociocultural awareness among the people concerned including healthcare providers and patients for active cooperation and participation to not only prevent patient safety accidents but also establish a reporting system that allows patients or caregivers to proactively check and communicate on patient safety. Furthermore, it is necessary to practice and provide training on participation in patient safety activities by including motivational strategies in the patient safety education program so that patients can actively participate. We laid the foundation for the transition research from medical personnel-centered research to general patients, and it is expected to contribute to creating a patient-centered patient safety culture through the application and development of more diverse educational methods in the future.

The limitations of this study are as follows: First, this study selected subjects by voluntary participation through a recruitment announcement for inpatients in a hospital, but there are restriction in sampling and selection of participants, so it is necessary to be careful in generalization. Second, there is a limitation in verifying the validity of the instrument that measures knowledge on patient safety due to lack of previous studies that were conducted on inpatients. Third, it was not possible to verify how the characteristics of the subjects’ underlying medical conditions, such as severity, pain, and surgery, affected the knowledge or awareness of patient safety. Fourth, there is a limit to conducting the survey on the day of hospitalization and the 5th or 7th day of hospitalization without considering the length of the subject’s stay in the hospital to confirm the effect of arbitration. Fifth, since a small number of hospitalized patients were conveniently extracted, there is a limitation that patient safety problems are unlikely to have been raised during the study period. Therefore, in future studies, it is necessary to ensure the generalization of the research results by recognizing these limitations, expanding the recruitment of subjects from more diverse medical institutions, and conducting repeated studies to provide a basis. Furthermore, we propose a study that increases the stability and feasibility of measurement tools that evaluate patient safety in patients. In addition, there is a need to develop a patient safety education program tailored to patients that considers the severity and medical characteristics (such as surgery status, inpatient duration) of their conditions and to verify the effectiveness of such a program.

## 7. Conclusions

In this study, there was significant improvement in the knowledge and awareness of patient safety in the experimental group that was exposed to the patient safety education program as compared to the control group that received safety education via printed educational materials. However, there was no significant difference in the willingness to participate in patient safety activities as both the experimental and control groups showed improvement. For the patient safety education program, video materials were developed using motion graphics and a tablet PC was used by the patients to repeatedly watch and easily access the videos from their beds. The results of this study revealed that the inpatients’ knowledge and awareness on patient safety were improved through access to related information. The study also confirmed the necessity of patients’ active participation in patient safety activities, for which opportunities should be provided through various means, and proper communication between the patients and healthcare providers should be encouraged.

### Clinical Implications

Since the education program developed in this study encouraged the subjects to repeatedly view the educational videos by inducing their interest, they were able to remember the contents for a long period of time; thus, these videos can be used as a useful medium for providing information. In future studies, it will be necessary to develop a standardized education program on patient safety that can be implemented using various educational media for people to easily access the content. Conducting a longitudinal study to determine how many patient safety incidents can be reduced when applying the patient safety education program to patients and caregivers would be worthwhile. Additionally, conducting a study with repeated measurements to evaluate knowledge and awareness on patient safety is also recommended. The patients’ willingness to participate should also be repeatedly evaluated, rather than evaluating the effectiveness of the education program just once.

## Figures and Tables

**Table 1:** Patient Safety Prevention Education.

Composition	Contents	Detail Contents	Time
1 video (7 min 38 s)	Understanding patient safety	Introduction of patient safety and laws	28 s
		Introduction of medical institution certification mark	26 s
		The importance of patient safety	34 s
	Patient safety activities	Patient identification	53 s
		Fall prevention	22 s
		Infection prevention	31 s
		Bedsores prevention	22 s
		Fire prevention	33 s
	Participation in patient safety (accurate communication)	Notify	51 s
		Check	64 s
		Ask	34 s
2 video (7 min 6 s)	Fall prevention activities	Raise and fix bed railing	25 s
		How to use a wheelchair	22 s
		Wear shoes that fit your feet	7 s
		Go to the bathroom before sleeping	10 s
		High risk patient management	13 s
	Infection prevention activities	Hand hygiene method	49 s
		When to perform hand hygiene	21 s
		Limited visitor and visit time	38 s
	Bedsores prevention activities	Skin condition check	31 s
		Change of position	
	Participation in patient safety (review)	Notify	42 s
		Check	
		Ask	
	Coping method in the patient safety problems	Introduction of the “Patient Safety Report Learning System”	40 s
		Introduction of the “Korea Medical Dispute Mediation Arbitration Agency”	

**Table 2:** Study design and procedure.

	Pre-Test	Post-Test	Pre-Test	Intervention		Post-Test
Period	20 July–11 September		20 September–13 November			
	HD 1	HD 5~7	HD 1	HD 3	HD 5	HD 7
Experimental group			E1, X1	X2	X3	E2
Control group	C1	C2				

HD: hospital day; E1, C1: patient safety knowledge, patient safety perception, willingness to participate in patient safety; X1: Treatment 1—first time video; X2: Treatment 2—second time video; X3: Treatment 3—watch the video repeatedly. E2, C2: Patient Safety Knowledge, Patient Safety Perception, Willingness to Participate in Patient Safety.

**Table 3:** Homogeneity test between experimental and control group (*N* = 69).

Characteristics	Categories	Total	Exp. (*n* = 33)	Cont. (*n* = 36)	χ^2^ or t	*p*
		*n* (%)	*n* (%) or M ± SD	*n* (%) or M ± SD		
Gender	Male	39 (56.5)	17 (43.6)	22 (56.4)	0.65	0.472
	Female	30 (43.5)	16 (53.3)	14 (46.7)		
Age group	20–39	23 (33.3)	10 (43.5)	13 (56.5)	1.10	0.576
	40–59	17 (24.6)	7 (41.2)	10 (58.8)		
	Over 60′s	28 (42.0)	16 (55.2)	13 (44.8)		
Final education	Middle school	19 (27.5)	9 (47.4)	10 (52.6)	0.89	0.956
	High school	26 (37.7)	13 (50.0)	13 (50.0)		
	University	24 (34.8)	11 (45.8)	13 (54.2)		
Economic level	High	5 (7.2)	4 (80.0)	1 (20.0)	2.57	0.276
	Middle	40 (58.0)	17 (42.5)	23 (57.5)		
	Low	24 (34.8)	12 (50.0)	12 (50.0)		
Religion	Yes	23 (33.3)	12 (52.2)	11 (47.8)	2.61	0.621
	No	46 (66.7)	21 (45.7)	25 (54.3)		
Job	Yes	47 (68.1)	21 (44.7)	26 (55.3)	0.58	0.606
	No	22 (31.9)	12 (54.5)	10 (45.5)		
Underlying disease	Yes	26 (37.7)	14 (53.8)	12 (46.2)	0.61	0.466
	No	43 (62.3)	19 (44.2)	24 (55.8)		
Medical institution visit	less than once a month	13 (18.8)	5 (38.5)	8 (61.5)	0.64	0.725
	less than once in 3 months	19 (27.5)	9 (47.4)	10 (52.6)		
	less than once a year	37 (53.6)	19 (51.4)	18 (48.6)		
Operation	Yes	45 (65.2)	22 (48.9)	23 (51.1)	0.06	1.000
	No	24 (34.8)	11 (45.8)	13 (54.2)		
Medical department	Medicine	41 (59.4)	19 (46.3)	22 (53.7)	3.49	0.175
	Surgery	22 (31.9)	13 (59.1)	9 (40.9)		
	Other	6 (8.7)	1 (16.7)	5 (83.3)		
Educational experience	Yes	55 (79.7)	29 (52.7)	26 (47.3)	2.61	0.139
	No	14 (20.3)	4 (28.6)	10 (71.4)		
Inpatient period			10.85 ± 8.20	8.92 ± 4.29	1.24	0.219
PS knowledge			22.88 ± 3.80	24.28 ± 3.13	−1.68	0.099
PS perception			97.24 ± 21.81	102.28 ± 20.52	−0.99	0.327
Willingness to participate in PS			61.21 ± 8.64	60.86 ± 10.73	0.15	0.882

PS = patient safety; Exp. = experimental group; Cont. = control group. *p* < 0.05.

**Table 4:** Differences of variables between the experimental and control group (*N* = 69).

Variables	Group	Pre-Test	Post-Test	Within Group		Difference	Between Group	
		M ± SD	M ± SD	*t*	*p*	M ± SD	*t*	*p*
PS knowledge	Exp. (*n* = 33)	22.88 ± 3.80	25.24 ± 1.98	3.94	<0.001	2.36 ± 3.44	2.52	0.014
	Cont. (*n* = 36)	24.28 ± 3.13	24.83 ± 3.03	1.35	0.187	0.56 ± 2.48		
PS perception	Exp. (*n*= 33)	97.24 ± 21.81	109.03 ± 9.95	3.78	0.001	11.79 ± 17.95	2.09	0.040
	Cont. (*n* = 36)	102.28 ± 20.52	105.44 ± 13.37	1.17	0.251	3.17 ± 16.27		
Willingness to participate in PS	Exp. (*n* = 33)	61.21 ± 8.64	65.85 ± 7.71	3.33	0.002	4.64 ± 7.99	0.71	0.478
	Cont. (*n* = 36)	60.86 ± 10.73	64.14 ± 9.42	2.51	0.017	3.28 ± 7.83		

PS = patient safety; Exp. = experimental group; Cont. = control group; *p* < 0.05.
